# AIDS patients family knowledge and behavior toward their patients before and after counseling

**DOI:** 10.1186/1742-4690-9-S1-P77

**Published:** 2012-05-25

**Authors:** Behnam Honarvar

**Affiliations:** 1 Communicable Diseases at Shiraz University of Medical Sciences, Shiraz, Iran

## Background

This study was aimed to measure the effect of ongoing counseling program at behavioral counseling center (BCC) of Shiraz, southern Iran on changing the knowledge ,attitude and behavior of AIDS patients' family members toward them.

## Methods and materials

125 members of HIV/AIDS patients’ family were interviewed individually by filling questionnaire before and after performing counseling for them. The findings were analyzed in SPSS.

## Results

The age of the participants was 40±13 years. Forty four percent had spousal relationships with their patients. Their knowledge about the main routes of HIV transmission were 9.76±2.59 and10.64±0.88 before and after counseling, respectively (P=0.028). Supportive behaviors of families toward their patients reached to 79 % after counseling compared with 44% before that (P=0.004). Belief to isolate the patients and the practice of this approach at home dropped from 71% to 15% and from 29% to 7% after counseling, respectively (P<0.05). In 30% of participants fear of getting HIV from patients was not changed by counseling, and 24% of patients’ spouses did report to avoid protected sex with their HIV infected husbands even after taking part in the counseling program (P>0.05).

## Conclusion

Ongoing counseling for HIV/AIDS patients’ families at BCC of Shiraz did advance their knowledge about AIDS and improved their attitude and behavior toward their patients. However, in some aspects such as the removal of fear about HIVspread in the family or the change of the patients’ wives attitude to have protected sex with their HIV infected husbands, the counseling program did not show remarkable success.

